# Sound Localization Training and Induced Brain Plasticity: An fMRI Investigation

**DOI:** 10.3390/diagnostics15121558

**Published:** 2025-06-18

**Authors:** Ranjita Kumari, Sukhan Lee, Pradeep Kumar Anand, Jitae Shin

**Affiliations:** 1 Electrical and Computer Engineering Department, Sungkyunkwan University, Suwon 16419, Republic of Korea; ranjita@skku.edu (R.K.); jtshin@skku.edu (J.S.); 2 School of Artificial Intelligence, Sungkyunkwan University, Suwon 16419, Republic of Korea; 3 Clinical Research Group, Samsung Healthcare, Seoul 05340, Republic of Korea; pradeep.ka@samsung.com

**Keywords:** functional MRI, neuroplasticity, sound localization, auditory system, vision system

## Abstract

**Background/Objectives:** Neuroimaging techniques have been increasingly utilized to explore neuroplasticity induced by various training regimens. Magnetic resonance imaging (MRI) enables to study these changes non-invasively. While visual and motor training have been widely studied, less is known about how auditory training affects brain activity. Our objective was to investigate the effects of sound localization training on brain activity and identify brain regions exhibiting significant changes in activation pre- and post-training to understand how sound localization training induces plasticity in the brain. **Method**: Six blindfolded participants each underwent 30-minute sound localization training sessions twice a week for three weeks. All participants completed functional MRI (fMRI) testing before and after the training. **Results:** fMRI scans revealed that sound localization training led to increased activation in several cortical areas, including the superior frontal gyrus, superior temporal gyrus, middle temporal gyrus, parietal lobule, precentral gyrus, and postcentral gyrus. These regions are associated with cognitive processes such as auditory processing, spatial working memory, planning, decision-making, error detection, and motor control. Conversely, a decrease in activation was observed in the left middle temporal gyrus, a region linked to language comprehension and semantic memory. **Conclusions:** These findings suggest that sound localization training enhances neural activity in areas involved in higher-order cognitive functions, spatial attention, and motor execution, while potentially reducing reliance on regions involved in basic sensory processing. This study provides evidence of training-induced neuroplasticity, highlighting the brain’s capacity to adapt through targeted auditory training intervention.

## 1. Introduction

As per the International Agency for the Prevention of Blindness (IAPB), approximately 43 million people were completely blind in 2020, which is projected to rise to around 61 million by 2050 [1]. Blind people face several challenges in performing daily tasks and interacting with their environment due to visual impairment.

Research on cross-modal plasticity and neural plasticity in individuals with visual impairment has shown that touch and sound are primary sensory modalities [2,3,4]. Touch facilitates detailed object perception, while sound enhances environmental awareness. Many researchers have studied the brains of visually impaired individuals using functional magnetic resonance imaging (fMRI) to understand how their brains adapt to different tasks. Advances in fMRI techniques have enhanced our understanding of the neural mechanisms underlying the heightened abilities of blind individuals, including superior performance in non-visual perceptual tasks [5], tactile discrimination [6], verbal memory [7], and sound localization [8,9]. In the lives of blind individuals, sound localization plays a pivotal role. Audio and tactile discrimination tasks have shown increased activation in the primary visual area (occipital cortex) of blind individuals [10,11], along with training-induced improvements in task performance, indicating neuroplasticity in the brain [12,13]. These studies suggest that sensory skill training in humans can result in alterations in the neural representation of the trained stimuli and that improvements in discrimination ability may be accompanied by more efficient processing in task-relevant brain areas. This type of learning-induced plasticity has been widely investigated in the sensory systems associated with the trained modality [14,15,16,17].

Furthermore, sound localization poses a unique challenge for the auditory system, relying on spatial acoustic cues generated by the interactions of sounds with the head and external ears. Spatial hearing depends on both binaural and monaural cues. The primary binaural cues encompass disparities in arrival time, referred to as interaural time difference (ITD), and variations in received intensity, known as interaural level difference (ILD). The predominant monaural localization cue involves alterations in the magnitude spectrum of sound due to its interaction with the head, body, and pinna before reaching the ear [18].

In spatial hearing investigations, positional representations of objects in three-dimensional (3D) space typically adhere to either Cartesian (x, y, z) or spherical (azimuth, elevation, distance) coordinates. An intuitive coordinate system for such studies is the bipolar spherical coordinate system, with the poles located at the two ears and the origin at the midpoint between them. In this system, azimuth, denoting the horizontal location of an object, is determined by the angle between the sound source and the interaural axis, while elevation, indicating the vertical location, is defined by the angle around the interaural axis. Distance is measured from the central point of the listener’s head [19]. This coordinate system is particularly conducive to discussions about spatial auditory perception.

Localization ability varies greatly among individuals and is often assessed using a sound localization acuity test [20]. Research has shown that sound localization can be improved through training. The human auditory system determines the spatial position of a sound source by processing auditory cues, a function critical for navigation, communication, and threat detection. This process involves the integration of neural activity across multiple brain regions, which collectively analyze acoustic signals to derive directional information.

While modifications in the human brain during sensory skill learning or training have not been fully investigated, and the neurobiological processes underlying training are poorly understood, evidence suggests that training-induced neuroplastic changes can occur [21]. Such evidence comes from behavioral studies focused on perceptual training, which have demonstrated improvements in sensory processing and associated neural adaptations. Several key studies contribute to this field, and some of the studies are discussed in the following paragraph.

An fMRI study investigated functional brain changes in blind and sighted adults following 10 weeks of click-based echolocation training. Post-training, both groups exhibited increased activation in the primary visual cortex (V1) in response to auditory echoes, demonstrating cross-modal neuroplasticity [22]. The right primary auditory cortex (A1) also showed heightened activation to sound stimuli, with blind participants exhibiting increased gray matter in right A1 and sighted participants showing changes in adjacent auditory regions. This expansion of the engaged neural networks after training reflects enhanced auditory, spatial, and sensorimotor processing, providing clear evidence of training-induced neuroplasticity.

Another study involving 15 congenitally blind individuals and 8 sighted individuals aimed to elucidate cross-modal plasticity in the blind population [23]. During tactile tasks, blind participants exhibited increased activation extending from the postcentral gyrus to the posterior parietal cortex, while a reduction in activity was observed in the secondary somatosensory area compared to their sighted counterparts.

In a separate study, 31 healthy individuals were divided into two groups: A Drum training group and a Control group, and observed for 8 weeks [24]. Training led to increased functional connectivity between the posterior superior temporal gyri, premotor and motor areas, and the right parietal lobe. Conversely, connectivity decreased within the cerebellum and certain temporal regions, suggesting that motor learning can reorganize resting-state brain networks.

An fMRI study of 18 participants found that sound recognition and localization tasks engage distinct neural networks [25]: Recognition primarily activated the bilateral middle temporal gyrus, precuneus, and left inferior frontal gyrus, whereas localization predominantly involved the inferior parietal lobule and posterior middle and inferior frontal gyri.

The above-related works conclude that a blind person exhibits very strong activation in the occipital lobe during the auditory task compared to a sighted person. These studies also reveal cross-modal plasticity. Our research seeks to investigate whether sound localization training induces neuroplasticity in the brain. Specifically, we aim to identify which brain regions, beyond the occipital lobe, exhibit significant neuroplasticity changes in response to sound localization training on blindfolded participants. To be precise, the following are our contributions.

- We performed fMRI on six participants before and after the sound localization training. Our research aimed to investigate, using fMRI, whether humans can improve sound localization after training and take advantage of neuroplasticity. We found that auditory adaptation is possible. Sound localization training can induce plasticity in the auditory localization system. Our results suggest that receiving feedback on disparities between actual and perceived locations of stimuli facilitates auditory spatial learning.
- We further investigated various regions of interest (ROI) and found that specific areas of the brain associated with the trained task or skill become more active or engaged. This increased activation often indicates neural plasticity, which is the brain’s ability to reorganize and form new connections in response to training and experience.
- Overall, our research findings support the idea that learning specific tasks can lead to neural adaptations, where individuals develop a more specialized and efficient neural network, particularly in regions responsible for movement, attention, memory, and spatial memory, to better handle the demands of the task.

## 2. Materials and Methods

In this section, we describe the participants, test setup, test procedure, data analysis, and statistical analysis used in the research.

### 2.1. Participants and Ethical Approval

Our research was conducted under the oversight of the IRB of the National Research Foundation of Korea. The IRB approval number is SKKU 2020-06-013, with the approval date of 24 July 2020. Six healthy right-handed adult participants (aged 23–30 years) were recruited for this study, comprising four males and two females. The inclusion criteria required that all participants should have normal hearing and have no history of neurological illness, as confirmed by their health check-up records. Information consent was obtained from all participants prior to the experiment, which was conducted in 2022.

### 2.2. fMRI Procedure and Scanning Parameters

**Figure 1:**
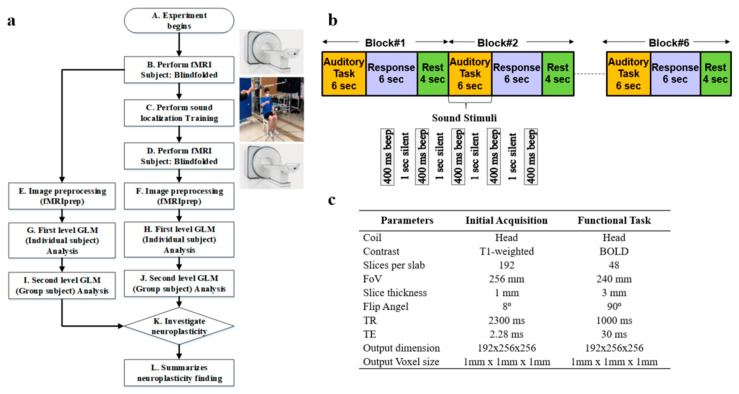
(**a**) fMRI experimental design. (**b**) Block diagram and structure of the stimuli for fMRI auditory and response task. (**c**) Experimental Pulse Sequence Parameters.

Whole-brain fMRI scan data were acquired using a 3T Siemens Prisma scanner (Oxford, United Kingdom) at Sungkyunkwan University, Suwon, South Korea. High-resolution T1-weighted structural images were also acquired, as shown in step B of Figure 1a. The scanner was equipped with magnetic-compatible KOSS headphones. Functional echo planner imaging (EPI) images were acquired with a repetition time (TR) of 460 ms, an echo time (TE) of 29.0 ms, a multiband acceleration factor of 8, a field of view of 256 mm, an 82 × 82 matrix, 3 × 3 × 3 mm^3^ voxels, and 56 interleaved slices. Stimulus presentation and behavioral data acquisition were controlled using MATLAB (3.7.2). A T1-weighted magnetic resonance (MR) image was first acquired to provide anatomical detail. The pulse sequence setting to acquire the routine anatomical image is tabulated in Figure 1c. We have used a field of view (FOV) of 256 mm with a slice thickness of 1 mm. The pulse sequence parameters for contrast setting are a repetition time (TR) of 2.3 s, a time to echo (TE) of 2.28 ms, and a flip angle of 8°. Then, a series of 128 gradient-echo blood-oxygenation-level-dependent (BOLD) images were acquired for the experiment with pulse sequence parameters as an FOV of 240 mm, a slice thickness of 3 mm, a TR of 1 s, a TE of 30 ms, and a flip angle of 90°. We used a sparse-sampling echo-planar imaging fMRI to avoid artifacts caused by scanning noise.

### 2.3. Sound Localization Training

Sound localization training was conducted on 6 blindfolded participants over 3 weeks, consisting of 6 sessions, as shown in step C of Figure 1a. Each week comprised 2 sessions, held on Tuesdays and Fridays. Within each session, there were five trials, and each trial involved sound from six distinct locations. At each location, sound stimuli were presented at 80 dB amplitude, 500 Hz frequency, 400 ms duration, and 1000 ms interval for 5 times. In this training, we included real-time sound-guided feedback and kinesthetic assistance. This feedback introduces more effective, guidance-based learning and promotes the development of well-honed human kinesthetic perception, leading to a better formation of neuroplasticity associated with 3D sound localization without visual aid. An article has been published by us showing the effectiveness of our sound localization training by improving the accuracy in terms of distance, elevation, and azimuth to locate the sound source accurately in 3D space [26].

After the sound localization training, fMRI was performed again in step D, the same as in step B of Figure 1a. We performed image preprocessing using fMRIprep for the image data collected before and after the sound localization training, as shown in steps E and F, respectively. Next, we conducted a first-level general linear model (GLM) analysis for each individual participant for the fMRI preprocessed data before and after sound localization training, respectively, in steps G and H as illustrated. Later, we performed second-level GLM analysis, which is also known as group-level analysis, for all 6 participants to discover common activation areas, as shown in steps I and J. Lastly, an investigation of the neuroplasticity of the brain to see the effect of sound localization training has been performed in step K of Figure 1a.

### 2.4. fMRI Task and Design Specification

Participants were checked to ensure they had no ferromagnetic materials on them before entering the MRI scan room. The participants then lay down on the table, and the head coil setup was performed by the experimenter. The table was moved up and slid in for the landmark experimenter to turn on the laser for the participant’s landmark. After that, the table was moved inside the bore to the home position, and the scan room was closed. Participants were asked to listen passively to the sounds while they were lying within the scanner. Auditory stimuli were presented binaurally over electrostatic MR-compatible KOSS headphones, and additional plastic muffs were added to attenuate the sounds of the scanner. The sound stimuli were presented at 80 dB, 500 Hz, with a 400 ms duration and 1000 ms interval, repeated 5 times as illustrated in Figure 1b. The participants were asked to close their eyes and listen carefully to the sound stimuli. We refer to this period as an auditory task, as illustrated in Figure 1b. After the auditory task, we communicated with the participants, instructing them to think about the location of the played sound and press the response button within 6 s. This period is referred to as the response task. We provided a 4 s rest period before repeating the auditory task and getting the response again.

A combination of auditory response and rest periods is referred to as one block of 14 s duration, as shown in Figure 1b. Each block onset was synchronized with the acquisition of the first brain image as soon as the first auditory task was initiated. We performed 6 blocks of tasks for each participant, as shown in Figure 1b. After finishing the scan, the test room was opened, and the experimenter slid the table out of the bore, lowered the table, removed the coil, and informed the participant that they could exit. Each participant went through the same scanning procedure.

### 2.5. fMRI Data Preprocessing

We performed preprocessing on fMRI image data for 6 participants before and after sound localization training using fMRIPrep. fMRIPrep [27] integrates cutting-edge techniques from leading preprocessing tools such as FMRIB Software Library (FSL), Advanced Normalization Tools (ANTs), FreeSurfer, and the Analysis of Functional Neuro Images (AFNI) to tackle various challenges inherent in fMRI data processing. The preprocessing pipeline includes motion correction, distortion correction, brain extraction, spatial normalization, and spatial smoothing. Notably, fMRIPrep automates the creation of anatomical and functional masks, streamlining subsequent analyses such as region-of-interest delineation and brain functional connectivity studies.

Preprocessing begins with the generation of a reference volume for accurate head motion correction. Head-motion parameters are estimated using MCFLIRT (FSL), and the BOLD reference is co-registered to the T1-weighted (T1w) reference using BBRegister (FreeSurfer). Confounding time-series metrics such as framewise displacement (FD) and DVARS are computed for each functional run, providing valuable insights into motion-related artifacts. Physiological regressors are extracted for component-based noise correction (CompCor), employing both temporal (tCompCor) and anatomical (aCompCor) approaches. Additionally, masks for cerebral spinal fluid (CSF) and white matter (WM) are generated for aCompCor, with careful consideration to exclude regions with minimal gray matter (GM) contribution.

Further, preprocessing steps involve the expansion of confound time series with temporal derivatives and quadratic terms to capture additional variability. Frames exceeding predefined thresholds for motion (0.5 mm FD or 1.5 standardized DVARS) are annotated as motion outliers. Nuisance time series are also derived from a principal component analysis of signals within a thin band of voxels around the brain’s edge. Resampling is performed with meticulous attention to detail, ensuring accurate alignment of data across different spaces using cubic B-spline interpolation.

### 2.6. First- and Second-Level Analysis

A program was written in Python 3.12.2 to perform first- and second-level statistical analysis on the preprocessed MRI images (output of fMRIprep). In the first-level analysis, we performed motion corrections to account for any movement during the scan, spatial normalization to align the brain images, spatial smoothing to improve the signal-to-noise ratio, and temporal filtering to remove low-frequency and high-frequency noise for each participant. We chose motion keys as translational and rotational for three axes, respective powers, their derivatives, and the power of derivatives. After motion correction, our code identifies brain regions that show a significant response after sound stimuli using GLM. In GLM, the brain’s response is modeled as a linear combination of explanatory variables representing our experimental conditions, along with motion parameters. We also visualized the result by developing an event and design matrix.

In the second-level analysis, our goal is to identify brain regions that consistently show a significant response across the six participants. We used *t*-tests for voxel-wise group comparisons to identify brain regions that are reliably involved in the activation during the response task. Furthermore, we used Neurosynth to understand the brain parts activated during the response task by uploading the fMRI images. The Neurosynth decoder provides a comparative Pearson correlation value for different brain regions activated by analyzing the fMRI images.

## 3. Results

Before presenting the fMRI results, we will briefly summarize the outcomes of the sound localization training. This sound localization training, incorporating sound-guided and kinesthetic feedback, was successfully conducted on six blindfolded participants over six sessions across three weeks. Each session, we performed the test for multiple trials to ensure participants had sufficient brain activation to process and estimate the sound source accurately. To minimize bias, six different test locations in 3D spaces were used in each trial. We found that all six participants showed significant improvement following the training. At the end of the training, distance, elevation, and azimuth errors were reduced by 73%, 80%, and 64%, respectively. Specifically, the mean distance error decreased from 574 mm to 152 mm, mean elevation error from 15° to 3°, and azimuth error from 28° to 10°. These results demonstrate the effectiveness of our sound localization training. A very comprehensive analysis of the results has been presented in our previously published article [26]. The fMRI results have been explained in two parts as a first (individual) and second (group) level of image analysis in the following sections.

### 3.1. First-Level Analysis Results

**Figure 2:**
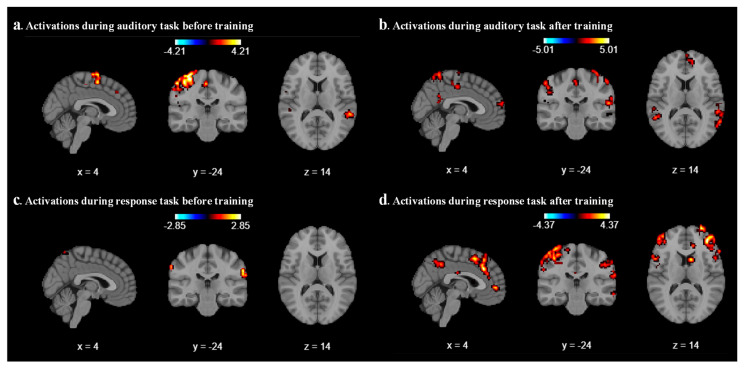
First-level GLM analysis of participant 2 with cluster size 10 and smoothing ON. (**a**) Middle temporal gyrus activated during auditory task before training. (**b**) Superior temporal gyrus and middle temporal gyrus activated during auditory task after training. (**c**) A mild activation in superior temporal gyrus during response task before training. (**d**) Higher activation seen in superior frontal gyrus and middle frontal gyrus during response task after sound localization training.

We analyzed the results for each participant by using the Nilearn package (0.10.4) in our code and performed first-level GLM analysis with various cluster sizes, smoothing on/off, and minimizing memory argument set to False. As a result of the first-level analysis, statistical maps such as t-maps representing brain activation for each participant have been produced during auditory and response tasks. We analyze the result with different combinations of cluster size, smoothing on/off, and different coordinates for each participant. After a few combinations, we realized that a cluster size of 10, with smoothing ON, provides a better contrast image for both auditory and response tasks before and after sound localization training across all six participants. As expected, we found that during the auditory task, the middle temporal gyrus and superior temporal gyrus areas were activated before and after localization training, as shown in Figure 2a and Figure 2b, respectively, for participant 2. For all other participants, different parts of the temporal lobes were activated during the auditory task. Figure 2c represents the contrast during the response task; a mild activation has been observed in the superior temporal gyrus. After the sound localization training, during the response task, strong activation was observed in the superior frontal gyrus and middle frontal gyrus, as shown in Figure 2d.

### 3.2. Second-Level Analysis Results

**Figure 3:**
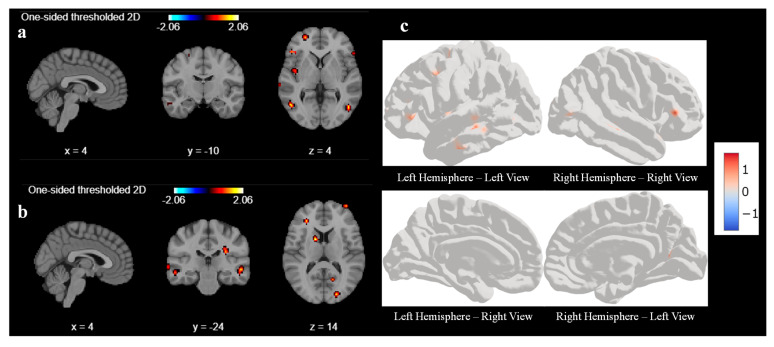
Second-level GLM analysis of all participants with cluster size 5 and smoothing ON. (**a**) Weak activation at superior frontal gyrus and middle temporal gyrus activated at coordinates (4, −10, 4) and (**b**) weak activations in the occipital gyrus and middle frontal gyrus at coordinates (4, −24, 14) during response task before sound localization training (**c**) Surface-based left and right sides of hemisphere of 3D brain.

As part of the second-level GLM analysis, our code uses the individual statistical maps from the first-level analysis as input and treats each participant’s data as a sample from the population. We use a one-sample *t*-test for our analysis. GLM second-level analysis is essential for advancing our understanding of brain function and its variability across individuals to draw more robust and generalizable conclusions for our study. The output of the second-level analysis indicates the areas of significant brain activation across the group, as shown in Figure 3. Like first-level analysis, we analyze the result with various cluster sizes and at different coordinates. With a cluster size of 10, there was no activation in any areas of the brain before the sound localization training during the response task. Hence, we reduced the cluster size from 10 to 5 to understand what part of the brain had been activated before the training during the response task. Figure 3a,b represent the output of the second-level GLM analysis with cluster size set as 5 before the training. We observed weak activations in the superior frontal gyrus and middle temporal gyrus at coordinate (4, −10, 4), as shown in Figure 3a, and a mild activation also seen in the occipital gyrus and middle frontal gyrus at coordinates (4, −24, 4) in Figure 3b. We tried to find out the activation by changing the coordinate, but we could not find any other significant signal. Figure 3c is a three-dimensional representation of the brain with active areas using surface-based datasets using second-level GLM for all 6 participants. Figure 3c represents the left and right hemispheres, respectively, of the brain image before sound localization training during the response period. There are very few active areas in the frontal lobes of the left and right hemispheres, which matches with second-level GLM results.

**Figure 4:**
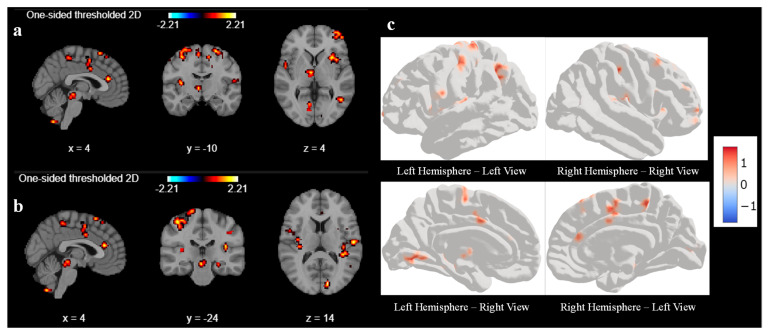
Second-level GLM analysis of all participants with cluster size 10 and smoothing ON. (**a**) activation in superior frontal gyrus, inferior frontal gyrus, precentral gyrus, postcentral gyrus, and middle temporal gyrus at coordinate (4, −10, 4) and (**b**) additional activation in the superior temporal gyrus, superior parietal lobules, and occipital lobe at coordinates (4, −24, 14) during response task after sound localization training; (**c**) Surface-based left and right sides of hemisphere of 3D brain.

Next, we performed a similar second-level GLM analysis during the response task after the sound localization training with a cluster size set as 10. We observed significant and strong activation in the different parts of the brain (unlike no activation before the training for cluster size 10), as shown in Figure 4. We observed higher activation signals in the superior frontal gyrus and inferior frontal gyrus in axial view in Figure 4a at coordinate (4, −10, 4). The superior frontal gyrus is involved in several higher cognitive functions, such as working memory and executive functions, which involve planning and decision-making. On the other hand, the inferior frontal gyrus is crucial for inhibitory control and attention, helping to suppress inappropriate responses and focus on relevant stimuli as cognitive control. We also observed high activation signals in the precentral gyrus and postcentral gyrus in the sagittal view, as shown in both Figure 4a,b. These areas are involved in motor-related activities and are vital for integrating and coordinating sensory inputs and motor outputs learned during the sound localization training, hence allowing for smooth and precise interactions with the environment.

The axial view of Figure 4a also shows a little activation in the middle temporal gyrus, which is responsible for memory as well as visual and auditory processing, which is one of the important parts of the sound localization training effect. Figure 4b shows similar activations as found in Figure 4a at coordinates (4, −24, 14). In addition, we also observed activation in the superior parietal lobules and occipital lobe in the axial view. Superior parietal lobules are responsible for spatial awareness and coordinating movements, thus enabling effective interaction with the environment. It works in conjunction with motor areas in the frontal lobe to coordinate precise and goal-directed actions, which plays an important role in sound localization training. Surface-based left and right sides of the hemisphere of the 3D brain image of second-level GLM analysis after sound localization training during the response task are shown in Figure 4c. Figure 4c shows very high activation in the precentral gyrus, postcentral gyrus, and superior parietal lobules of the left hemisphere during the response task after the sound localization training. In addition, we can also see activation in the frontal area of the right hemisphere. These activated areas reconfirm our findings as explained in Figure 4a,b.

To ensure the reliability of the GLM analysis results, we performed statistical analyses to calculate the family-wise error rate (FWE) and false discovery rate (FDR) before and after the training. We applied stringent test conditions with a significant threshold of *p* < 0.05 and used the highly conservative Bonferroni correction. The FWE and FDR values were 4.793 and 4.930, respectively, before training, in comparison to 4.800 and 4.938 after training, based on the second-level GLM analysis. These results indicate that only extremely robust brain activations reached statistical significance after correcting for multiple comparisons, with both FWE and FDR thresholds exceeding 4.8.

Furthermore, we performed the contrast analysis to reconfirm our findings during the second-level GLM analysis. Contrast analysis in fMRI studies is a statistical technique used to identify brain regions that exhibit significant changes in activity under two different conditions. Considering this, we performed a paired two-sample *t*-test to compare brain activation before and after sound localization training, allowing us to quantify training-induced neuroplasticity. Contrast analysis helped us to isolate task-specific neural changes by subtracting baseline (pre-training) activation from post-training activation, thereby highlighting regions where significant differences were observed. By applying this contrast analysis, we were able to determine which cortical areas showed increased or new activation in response to sound localization training, minimizing inter-participant variability and enhancing the statistical power of our findings.

**Figure 5:**
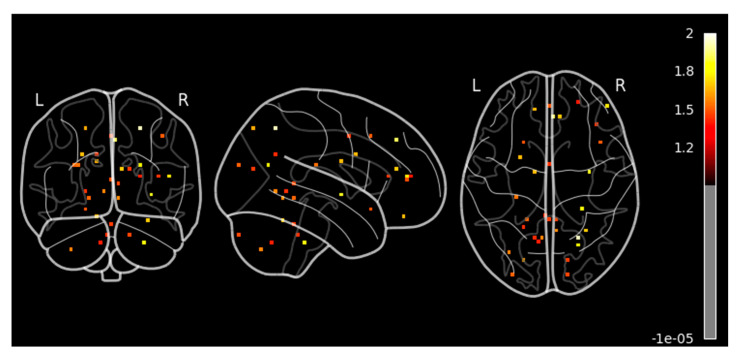
Contrast analysis of all participants with cluster size 10 and smoothing ON. A high activation seen in superior frontal gyrus and superior parietal lobule, and weak activation observed in middle frontal gyrus in sagittal view. High activation observed in superior frontal gyrus, right middle temporal gyrus, and right parietal lobule in the axial view.

Our results revealed significant activation in the superior frontal gyrus and superior parietal lobule in sagittal view, as shown in Figure 5 and summarized in Table 1. There was mild activation in the precentral, postcentral, and middle frontal gyrus. In the axial view, we observed a high activation in the superior frontal gyrus, right middle temporal gyrus, and right parietal lobule. These regions are known to be involved in attention, spatial cognition, motor execution, sensory processing, and auditory perception. In this context, the method provides valuable insights into the cortical reorganization associated with improved auditory-motor integration, reinforcing the idea that sound localization training can drive neuroplastic adaptations in the human brain.

## 4. Discussion

The primary aim of this study is to identify the brain regions activated before and after sound localization training and to determine if such training induces neuroplasticity in the human brain. In our study, we observed both increased and decreased activation in various brain regions following sound localization training. Specifically, functional neuroimaging studies have shown that the spatial and temporal distribution of brain activity can differ significantly before and after a targeted training regimen. These changes in activation patterns are often attributable to the specific cognitive or perceptual demands imposed by the training task. Increased activation was noted in areas such as the superior frontal gyrus, superior temporal gyrus, right middle temporal gyrus, and parietal lobule, indicating the engagement of higher-order cognitive and spatial processing networks essential for acquiring and refining sound localization skills, as detailed in Table 1. Some other studies also found that sound localization activates a right-lateralized network including the posterior superior temporal gyrus, inferior parietal lobule, and premotor cortex—regions implicated in spatial computations and sensorimotor integration [28,29]. We also observed a mild activation in the precentral gyrus and postcentral gyrus. These areas are linked to motor planning and integration of multisensory information and information manipulation.

Conversely, we observed a decrease in left middle temporal gyrus activation after training, a region associated with language and semantic processing that may reflect reduced reliance on compensatory or less efficient strategies, consistent with the shift from ventral to dorsal (spatial) processing.

The superior frontal gyrus aids sound localization training by enhancing attention and focus, supporting episodic memory, which holds and manipulates spatial information about sound sources, and aiding accurate localization [30,31]. An increase in activation was also observed in the superior temporal gyrus. The superior temporal gyrus is crucial for processing auditory information, including the perception of sound frequency, intensity, and timbre, which is related to sound-guided feedback provided during sound localization training. Prior to training, activation is observed bilaterally in the middle temporal gyrus. However, after training, activation in the left middle temporal gyrus is no longer evident. The right middle temporal gyrus is responsible for spatial and auditory attention. Some other studies also found that before training, the superior temporal gyrus was activated bilaterally; after training, the activation was reduced in the right superior temporal gyrus [32]. This shift in activation suggests that the auditory-spatial areas become more engaged following training, likely reflecting the increased reliance on auditory-spatial integration for sound localization.

The superior parietal lobule plays a critical role in sound localization training through its involvement in spatial monitoring, sensory-motor integration, and auditory working memory. During the sound localization training, tracking sound movement in space receives input from the auditory and somatosensory systems and integrates them for spatial awareness. This integration is essential for localizing sound sources in complex environments where participants have to identify 3D sound position, especially when visual cues are absent (e.g., in blindfolded and blind individuals). Some studies also found that the right parietal lobule showed significant activation when participants processed moving sounds [33,34].

The activation of the precentral gyrus indicates the involvement of motor areas in the training task. This observation aligns with the nature of the task, as participants were required to perform rapid aiming movements during each trial. They integrate auditory information with motor actions, adjusting motor responses based on the auditory feedback and enhancing training effectiveness. The postcentral gyrus activation indicates that during sound localization training, it helps to integrate auditory cues with other sensory inputs, thus providing a comprehensive spatial map of the environment.

After training, when the participants are localizing the sound source they hear, they try to link the multimodal sound localization skill they learned and memorized during training, resulting in the activation of several brain regions involved in motion, memory, and spatial mapping. Whether or not such a spread of brain activations is leading to a sustainable change in plasticity remains to be further investigated. However, our findings may open a new possibility of training humans for its multimodal perceptual integration with sustainable change in brain plasticity. These neuroplastic changes provide a scientific basis for developing structured auditory training protocols in the rehabilitation of individuals with visual impairments. Such interventions could be integrated into orientation and mobility training programs, potentially augmented with multisensory feedback or assistive technologies, to enhance spatial awareness, navigation skills, and overall functional independence in the visually impaired population.

We conducted a comparative study of our research with similar studies on induced neuroplasticity as listed in Table 2. We could not find any research on sound localization training that investigated neuroplasticity in blind or blindfolded participants. It has been found that there is increased functional connectivity in the premotor and motor regions after drum training conducted over eight sessions [24]. However, motor drum training was performed on sighted individuals, unlike the blindfolded participants in our study. In another study, it was found that the inferior parietal lobule and the posterior parts of the middle and inferior frontal gyri were more activated during the localization task [25]. However, in this research, there was no training, and the sound localization task was conducted on sighted individuals.

We would like to highlight the limitations of our research. The number of participants was six. Due to limited research funding and the unavailability of blindfolded participants, we initially planned for eight participants. Later, two participants dropped out of the study as they found it difficult to commit to at least eight sessions (initial fMRI, six days of sound localization training over a three-week duration, and final fMRI after training). As five out of six participants performed equally well during this study, we believe the research outcomes are valid even with a smaller sample size. We also acknowledge that the absence of a non-training control group is a limitation of our current study. To mitigate this, we implemented a within-participants pre- and post-training design, allowing each participant to serve as their own control. This approach enabled us to identify significant changes in brain activation patterns attributable to the training regimen. In Phase 3 of our future research, we plan to include a control group—such as one comprised of participants exposed to no training at all—to provide a basis for better distinguishing training-specific effects from potential confounding factors such as the scanning environment or time-related changes.

Another limitation of this study was that sound localization training and fMRI were conducted on blindfolded instead of blind participants. Recruiting blind participants for an fMRI study is highly challenging, as many blind participants are uncomfortable in the confined space of the MRI bore. Additionally, this experiment was conducted during the COVID-19 pandemic, which further complicated the recruitment process. However, it has been proven by several prior studies that blind individuals outperform blindfolded individuals in auditory tasks. Therefore, our assumption is that we would obtain better results from blind individuals for sound localization training and neuroplasticity studies using fMRI.

## 5. Conclusions

Sound localization is a complex process that involves the integration of information across multiple brain regions, each specialized for different aspects of auditory processing and spatial perception. Our finding is that sound localization training induces plasticity in the human brain, as evidenced by increased activation in regions superior frontal gyrus, superior temporal gyrus, right middle temporal gyrus, superior parietal lobule, precentral gyrus, and postcentral gyrus, which are involved in cognitive processing, working memory, motor control, and multisensory integration. The engagement of multiple neural systems highlights the complex nature of the training task and its impact on brain function due to kinesthetic and sound-guided feedback provided during sound localization training. As future work, we plan to extend our research using a multimodal approach—combining MRI and electroencephalogram (EEG)—in a phase 3 study with an increased number of participants. While the within-participants design in the current study allowed us to observe training-related changes, we acknowledge that including a non-training control group in future studies will strengthen the ability to attribute observed neural adaptations specifically to sound localization training.

## Figures and Tables

**Table 1:** Activated areas before and after training.

Coordinate	Before Localization Training	After Localization Training
X = 4 Y = −10 Z = 4	Middle frontal gyrus Inferior frontal gyrus Head of caudate nucleus Middle temporal gyrus (Left and Right)	Superior frontal gyrus Inferior frontal gyrus Precentral gyrus Postcentral gyrus Middle temporal gyrus (Right) Superior temporal gyrus
X = 4 Y = −24 Z = 14	Occipital lobe	Superior parietal lobules Occipital lobe

**Table 2:** Comparative study with similar study for induced neuroplasticity.

Research Title	Training/Task	Session	Feedback	Method	Participants	Activated Parts During fMRI
Sound localization training and Induced Brain Plasticity—An fMRI Investigation	Sound localization perceptual	6 sessions 5 trials/session 30 min/session	Sound-guided and kinesthetic	fMRI	Blindfolded	Higher activation observed in pre- and post-central gyrus, superior and middle temporal gyrus, and superior frontal gyrus post sound localization training
Motor learning induces Plasticity in the resting brain [24]	Drum playing	8 sessions 30 min/session	No feedback	fMRI	Sighted	Increased functional connectivity in the premotor and motor regions after drum training
Distinct pathway Involved in sound recognition and localization [25]	No training Sound localization and recognition task	Not applicable	No feedback	fMRI	Sighted	More activation in inferior parietal lobule and the posterior parts of the middle and inferior frontal gyri during sound localization task

## Data Availability

The data supporting the findings of this study are available upon reasonable request from the corresponding author, S.L.

## Abbreviations

The following abbreviations are used in this manuscript:

fMRI	Functional magnetic resonance imaging
ITD	Interaural time difference
ILD	Interaural level difference
FC	Functional connectivity (of brain)
ROI	Region of interest
IRB	Institutional review board
SKKU	Sungkyunkwan University
EPI	Echo planner imaging
TR	Repetition time
TE	Echo time
BOLD	Blood oxygenation level dependent
GLM	General linear model
KOSS	A brand name of headphone from Koss Corporation
FSL	FMRIB software library
MCFLIRT	Motion correction using FMRIB’s linear image registration tool
BBRegister	Boundary-based registration
FD	Framewise displacement
CompCor	Component-based noise correction
CSF	Cerebral spinal fluid
WM	White matter
GM	Gray matter
DVARS	D temporal derivative of time courses, VARiance across voxels
FMRIB	Functional MRI of the Brain
ANT	Advanced Normalization Tools
AFNI	Analysis of Functional Neuro Images
FEW	Family-wise error rate
FDR	False discovery rate
